# Health-Related Quality of Life among Children with Recurrent Respiratory Tract Infections in Xi’an, China

**DOI:** 10.1371/journal.pone.0056945

**Published:** 2013-02-25

**Authors:** Xun Jiang, Lijun Sun, Baoxi Wang, Xianjun Yang, Lei Shang, Yuhai Zhang

**Affiliations:** 1 Department of Pediatrics, Tangdu Hospital, Fourth Military Medical University, Xi’an, Shaanxi, China; 2 Department of Health Statistics and the Ministry of Education Key Lab of Hazard Assessment and Control in Special Operational Environment, School of Public Health, Fourth Military Medical University, Xi’an, Shaanxi, China; Old Dominion University, United States of America

## Abstract

**Objective:**

The aim of this study was to investigate the health-related quality of life (HRQOL) in 2−7-year-old children diagnosed with recurrent respiratory tract infections (RRTIs) and the impact of RRTIs on affected families.

**Methods:**

This was a cross-sectional case-control study evaluating 2−7-year-old children with RRTIs (n = 352), 2−7-year-old healthy children (n = 376), and associated caregivers (parents and/or grandparents). A Chinese version of the PedsQL™ 4.0 Generic Core Scale was used to assess childhood HRQOL, and a Chinese version of the Family Impact Module (FIM) was used to assess the impact of RRTIs on family members. HRQOL scores were compared between children with RRTIs and healthy children. In addition, a multiple step-wise regression with demographic variables of children and their caregivers, family economic status, and caregiver’s HRQOL as independent variables determined factors that influenced HRQOL in children with RRTIs.

**Results:**

Children with RRTIs showed significantly lower physical, emotional, social, and school functioning scores than healthy children (*p*<0.05). Caregivers for children with RRTIs also scored significantly lower than caregivers for healthy children on physical, emotional, social, cognitive, and communication functioning (*p*<0.05). Caregivers for RRTIs affected children also reported significantly higher levels of worry. Multivariate analyses showed that children’s age, children’s relation with caregivers, the frequency of respiratory tract infections in the preceding year, caregiver’s educational level, and caregiver’s own HRQOL influenced HRQOL in children with RRTIs.

**Conclusions:**

The current data demonstrated that RRTIs were associated with lower HRQOL in both children and their caregivers and negatively influenced family functioning. In addition, caregivers’ social characteristics also significantly affected HRQOL in children with RRTIs.

## Background

Recurrent respiratory tract infections (RRTIs) are one of the most commonly occurring diseases [1]. Importantly, RRTIs are the most prevalent disease among children, especially for 2−6-year-old children, and RRTI incidence is approximately 20% in mainland China [2]. RRTIs are often related to genetic factors, lower immune functioning, vitamin and mineral deficiencies, improper feeding and nursing, residential environment, and other variables. If not properly treated, RRTIs can lead to asthma, myocarditis, nephritis, and/or other diseases, which may seriously affect a child’s growth and development [2]. Therefore, it is important that RRTIs be studied in pediatric practice.

Health-related quality of life (HRQOL) is an essential health outcome measure used in clinical trials and health service research and evaluation [3]. A HRQOL assessment may identify unexpected functional disability, monitor disease progression, and improve patient prognosis. Nevertheless, it is estimated that pediatricians’ apply practical use of HRQOL information in clinical decision-making in less than 25% of cases [4]. Although interest in HRQOL measurements and biomedical indicators for pediatric health outcomes has grown in recent years [5], so far the assessments have been limited to specific diseases that are unrelated to RRTI’s [6].

Increased risk for RRTIs has considerable impact on a child’s physical functioning and family economy, including lost working days of caregivers and other family-related functions [7]. To our knowledge, research has yet to evaluate HRQOL in children with RRTIs and their family members; however, RRTIs likely affect both children and their caregivers’ quality of life. Therefore, the purpose of this study was to assess the differences in HRQOL between children with RRTIs and healthy children, as well as to describe the impact of RRTIs on caregivers and/or family members. This research aimed to provide scientific insight into ways to address and improve HRQOL in children with RRTIs and their caregivers.

## Materials and Methods

### RRTIs Diagnostic Criteria

The main diagnostic criteria for childhood RRTIs included the following guidelines [8]: the child experienced at least six (if ≥3 years of age) or eight (if <3 years of age) practitioner-attended respiratory tract infections during the preceding year. Pediatric outpatient medical records defined RRTI episodes for each participant. In addition, these medical records provided a child’s sociodemographic characteristics and medical and/or health care history (diagnosis, treatment, examination, etc.) as recorded by a pediatrician.

### Data Source

Between January 2011 and July 2011, 352 children (2−7 years) with RRTIs and their caregivers (parents or grandparents) were selected from the Pediatric Outpatient Departments at Xijing hospital and Tangdu hospital in Xi’an, China and comprised the patient group. The healthy control group included 376 healthy children (2−7 years old) and their caregivers (parents or grandparents) selected from the children’s health care centers at the same two hospitals where the patient group participants were enrolled. Healthy children were all being seen for a routine annual well check. All subjects had an outpatient medical and health care record at one of the above mentioned hospitals, and caregivers confirmed that the selected hospital was the first medical or health care institution for the child.

The selected hospitals are two of the largest AAA Hospitals in Xi’an. The AAA distinction means that they are among the best hospitals in China with the capacity to provide high-level medical services and implement high-level medical education and research projects. One of the hospitals is located in the urban center of Xi’an and the other is located in the suburbs of Xi’an. Both hospitals are available for the use by the whole population of Xi’an and both treat a comprehensive spectrum of diseases. The pediatrics departments of the two hospitals together service more than 600 patients per day, and each has more than 200 beds for admitting children. Both departments are further divided into several sub-specialties, including respiratory diseases, digestive diseases, kidney diseases, and cardiovascular diseases.

For this study, patient group criteria included the following: 1) age 2−7 years old, 2) medical records at one of the designated study hospitals, 3) pediatric-diagnosed RRTIs, 4) nursery school or kindergarten attendance, and 5) during the preceding year, only study designated hospitals were used for health care/consultation. Child subjects were excluded from the patient group if they had any other medical condition, including chronic diseases (e.g. asthma, heart diseases, renal disease, cancer, or epileptic), neurological developmental disorders(e.g. autism spectrum disorder, dyspraxia, conduct disorder), or growth abnormalities (e.g. dwarfism, failure to thrive, etc.) ,as such conditions may influence HRQOL. Subjects were also excluded if their caregivers were illiterate or reluctant to participate.

Participation of healthy control group subjects depended on the following criteria: 1) age 2−7 years old, 2) health care records located at one of the designated study hospitals, 3) diagnosed by a pediatrician as developing normally and being healthy and free from respiratory infections for the past 6 months, 4) nursery school or kindergarten attendance, and 5) during the preceding year, only study designated hospitals were used for health care/consultation. The same exclusion criteria applied to the patient group were also applied to the control group (chronic diseases, developmental disorders, growth abnormalities, and illiterate or noncompliant caregivers were excluded). In addition, children who received functional therapy for a disability or nutritional counseling for specific medical conditions were also excluded.

The Research Ethics Committee of the Fourth Military Medical University approved this study. All caregivers provided written informed consent prior to the collection of any information.

### HRQOL Assessment

A Chinese version of the Pediatric Quality of Life Inventory™, version 4.0 (PedsQL^TM^4.0) was used in this study. The PedsQL^TM^4.0 consisted of the following three parts:

#### (1) The pedsQL™ 4.0 generic core scales

The PedsQL™ 4.0 Generic Core Scale was developed by Varni et al [9], [10], [11]. This scale has been cross-culturally adapted into Chinese by Hao et al [12] and demonstrates good internal consistency, as well as discriminant and construct validity [12], [13]. The Generic Core Scale is a brief, 23-item standardized questionnaire assessing a pediatric patient’s HRQOL, such that HRQOL is operationalized as the perceived impact of disease and treatment on a variety of functional domains (physical, emotional, social, and school functioning) [9], [10]. Parents proxy-report and children self-report the degree that each item has been a problem for the child during the past month using a 5-level scale: 0 = never a problem, 1 = almost never a problem, 2 = sometimes a problem, 3 = often a problem, and 4 = always a problem. Items were then linearly transformed to a 0−100 scale (0 = 100, 1 = 75, 2 = 50, 3 = 25, 4 = 0), so that higher scores indicated better HRQOL. The dimension scores were computed as the sum of the items divided by the number of items answered within a particular dimension.

#### (2) The pedsQL™ 4.0 family impact module (FIM)

The PedsQL™ 4.0 FIM was developed by Varni et al [9], [14] and was cross-culturally adapted into Chinese by Chen et al [15]. The module demonstrates good internal consistency, as well as discriminant and construct validity. The FIM is a parent-reported instrument that measures the impact of pediatric chronic health conditions on patient HRQOL and their family functioning. This 36-item instrument consists of 8 dimensions: physical functioning (6 items), emotional functioning (5 items), social functioning (4 items), cognitive functioning (5 items), communication (3 items), worry (5 items), daily Activities (3 items), and Family Relationships (5 items) [14]. The first six dimensions measure parent self-reported functioning, while the latter two dimensions measure parent-reported family functioning. Likert-type scale responses are provided for each item: 0 = never a problem, 1 = almost never a problem, 2 = sometimes a problem, 3 = often a problem, and 4 = almost always a problem. Items are then linearly transformed to a 0−100 scale (0 = 100, 1 = 75, 2 = 50, 3 = 25, 4 = 0), which means that higher scores indicate better HRQOL.

The FIM obtains three types of summary scores: 1) The total score is calculated as the sum of all 36 items divided by the number of items answered; 2) the parent HRQOL summary score is calculated as the sum of the 20 items from Physical, Emotional, Social, and Cognitive Functioning dimensions divided by the number of items answered; 3) the Family Functioning Summary Score is calculated as the sum of the 8 items from Daily Activities and Family Relationship dimensions divided by the number of items answered.

#### (3) PedsQL™ family information form

The PedsQL™ Family Information Form was also developed by Varni et al [9], [10]. This form has been cross-culturally adapted into Chinese and contains a child’s basic information (date of birth, gender, disease duration) and a caregiver’s basic information (marital status, occupation, family income level, and method of payment for the child’s medical care). In addition, a caregiver’s educational status and the relation between child and his/her caregiver were items added to this form.

### Data Collection and Quality Control

Five pediatric nurses, with at least 5 years of pediatric clinical nursing experience, performed this study. All of the investigators were trained prior to survey administration in order to ensure they had mastered the survey purpose and its requirements. Caregivers completed the PedsQL™ 4.0 Generic Core Scales for 2−4 year old children,whereas caregivers and 5−7 year old children completed this survey. The children’s caregivers answered The PedsQL™ 4.0 FIM and PedsQL™ Family Information Form.

An investigator explained the purpose and details of all scales so that both the caregivers and children could successfully complete the assessments. In addition, one of the main investigators carefully rechecked all the scales, and telephone interviews were conducted for any lost or incomplete information. EpiData 3.1 software was used for data entry. To ensure data accuracy, double entry mode was selected and logistical errors were corrected.

### Statistical Analysis

Quantitative data was expressed as 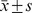
_,_ and qualitative data was expressed as a percentage value. A Chi-square (*χ* ^2^) test compared the distribution of children’s gender, their caregiver’s gender, their caregiver’s education level, the relation between the child and his/her caregiver, and family economic status. Independent sample *t*-tests determined differences in age, Generic Core Scales, and FIM scores between the two groups (RRTIs vs. healthy). A paired *t*-test compared Generic Core Scale score differences between self-reports and caregiver proxy-reports. Pearson correlation coefficients (*r* values) were calculated to determine the relationship between Generic Core Scale scores and FIM scores. Bonferroni corrections were applied to control for multiple testing. Multiple step-wise regression analysis evaluated factors that influenced the HRQOL in children with RRTIs. Specifically, the influences of demographic characteristics (both child and caregiver), child-caregiver relationship, frequency of respiratory tract infections in the preceding year, and caregiver’s HRQOL scores on Generic Core Scale dimensions were determined. Statistical significance was accepted at *p*<0.05. All analyses were performed using the Statistical Package for Social Sciences software program, version 16 (SPSS, Inc., Chicago, IL, USA).

## Results

Child and caregiver demographics are reported in Table 1. Neither gender nor age differed significantly between children with RRTIs and healthy children (*p*>0.05). In addition, the proportion of parents among caregivers, caregiver’s education level, and family economical status did not differ between the two groups (*p*>0.05).

**Table 1:** Child and caregiver demographic information.

	Children with RRTIs (n = 352)	Healthy children(n = 376)
Children’s gender(boys,n(%))	196(55.7)	204(54.3)
Children’s age( 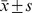 , years)	5.2±2.3	5.3±1.9
Relation between child and Caregiver		
Parents, n(%)	217(61.7)	235(62.6)
Grandparents, n(%)	135(38.3)	141(37.4)
Caregiver’s education level(n(%))		
≤9 years	80(22.7)	96(25.5)
9–12 years	124(35.2)	108(28.7)
≥13 years	148(42.1)	172(45.3)
Family economic status (n(%))		
≤1500 yuan/individual·month	84(23.9)	100(26.6)
1500 ∼3000 yuan/individual·month	140(39.8)	144(38.1)
≥3000 yuan/individual·month	128(36.3)	132(35.1)

Table 2 presents the internal reliability estimates (Cronbach’s alpha coefficients) of the Generic Core Scale and FIM Scale summaries and subscales. The internal consistency estimate for the General Core Scale summary was 0.89. Subscale coefficients ranged from 0.74 to 0.83. For the FIM scales, internal consistency estimate of the summary scale was 0.91, and subscale coefficients ranged from 0.82 to 0.89. Moderate to high item-summary correlations were observed across the scales.

**Table 2:** Internal consistency (Cronbach’s alpha) and item-summary correlations between the PedsQL™ 4.0 Generic Core Scale and FIM Scale.

Dimension	Cronbach’s α coefficient	Item-summary correlation
**Generic Core Scale**		
Physical	0.83	0.51–0.69
Emotional	0.81	0.56–0.67
Social	0.76	0.48–0.67
School	0.74	0.45–0.62
Summary scale	0.89	0.54–0.68
**FIM**		
HRQOL		
Physical	0.89	0.60–0.82
Emotional	0.87	0.57–0.76
Social	0.85	0.56–0.84
Cognitive	0.88	0.56–0.71
Communication	0.83	0.46–0.67
Worry	0.85	0.61–0.68
Family Functioning		
Family daily activities	0.82	0.57–0.78
Family relationships	0.89	0.56–0.73
Summary scale	0.91	0.52–0.78

Table 3 lists caregiver proxy-report scores for the Generic Core and FIM Scales. Children with RRTIs reported significantly lower scores on every dimension of the Generic Core Scale compared to healthy children (*p*<0.05). Emotional dimension scores differed the most between the two groups. Physical functioning, social functioning, school functioning and summary scale scores were also significantly lower in 5−7-year-old children with RRTIs compared to 2−4-year-old children with RRTIs (*p*<0.05), and of the four dimensions, physical functioning scores differed most.

**Table 3:** Caregiver proxy-report mean scores (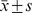) on the Generic Core Scale and FIM Scale.

	2∼4 years olds		5∼7years olds	
	healthy(n = 200)	RRTIs(n = 220)	Healthy(n = 152)	RRTIs(n = 156)
**Generic Core Scales score**				
Physical	92.6±6.8	84.2±12.2^ab^	93.8±8.7	69.8±19.6^a^
Emotional	88.7±13.3	63.4±13.7^a^	88.1±10.7	62.8±14.7^a^
Social	95.4±8.0	79.7±13.0^ab^	95.5±9.9	74.6±15.0^a^
School	89.5±8.0	67.9±7.1^ab^	88.5±16.0	61.7±13.3^a^
Summary scale	92.1±6.7	75.5±7.6^ab^	93.2±9.2	67.6±12.9^a^
**FIM score**				
HRQOL	88.8±8.0	63.3±8.3^a^	88.5±9.4	64.8±9.2^a^
Physical	89.4±12.8	64.5±11.4^a^	91.5±9.8	65.3±12.2^a^
Emotional	92.6±9.2	63.2±12.6^a^	93.5±9.5	65.4±13.4^a^
Social	88.9±14.0	61.5±12.9^a^	91.2±12.3	65.5±17.7^a^
Cognitive	87.7±16.3	63.6±13.7^a^	85.6±16.3	64.5±13.7^a^
Communication	94.4±8.9	73.5±15.9^ab^	90.4±11.4	68.0±17.9^a^
Worry	57.1±18.2	81.9±17.5^a^	61.5±18.3	79.6±17.7^a^
Family Functioning	85.7±12.8	62.0±12.9^ab^	85.1±13.9	66.8±13.7^a^
Family daily activities	83.0±19.2	55.8±16.9^ab^	80.8±18.8	62.4±17.8^a^
Family relationships	87.4±14.1	65.7±16.3^a^	87.6±14.7	69.3±14.6^a^
Summary scale	87.9±7.7	62.9±8.4^a^	87.4±9.8	65.0±9.7^a^

a *p*<0.05, RRTI children vs. age-matched healthy children;

b *p*<0.05, 2−4 year old children with RRTIs vs 5−7 year old children with RRTIs.

Children with RRTSs in both age bands had significantly lower FIM scores than those of healthy children on every dimension except the worry dimension (*p*<0.05). Moreover, the greatest difference between scores of caregivers of children with RRTIs and scores of caregivers of healthy, similarly aged children was in the emotional function dimension. Communication score was significantly higher in 2–4 year-old children with RRTIs than in 5-7-year-old children with RRTIs. However, family functioning and family daily activities scores were significantly lower in 2−4-year-old children with RRTIs than in 5−7-year-old children with RRTIs (*p*<0.05).

Table 4 shows the self-reported Generic Core Scale scores for 5−7-year-old children with RRTIs and their caregiver proxy-report scores. Children with RRTIs self-reported significantly higher physical, emotional, school and summary scale scores than their caregiver’s reported in proxy-reports (*p*<0.05). The social dimension score of children and caregiver were similar (*p*>0.05).The emotional functioning self-report scores among healthy children were significantly higher than their caregiver proxy-report scores, while the opposite pattern was observed for summary scores (*p*<0.05).

**Table 4:** Child (5−7 years) self-report and caregiver proxy-report PedsQL™ Generic Core Scale scores.

Score	Children with RRTIs (n = 156)		Healthy children (n = 152)	
	Caregiver proxy-report	Child self-report	Caregiver proxy-report	Child self-report
Physical	69.8±19.6^a^	76.4±18.2	93.8±8.7	93.8±8.2
Emotional	62.8±14.7^a^	69.0±17.6	88.1±10.7^b^	92.3±8.7
Social	74.6±15.0	78.4±15.2	93.5±9.9	93.3±12.6
School	61.7±13.3^a^	68.2±16.8	78.5±15.7	77.7±19.4
Summary	67.6±12.9^a^	73.5±12.3	89.7±8.7^b^	78.4±13.2

a *p*<0.05, RRTI child self-report vs. caregiver proxy-report;

b *p*<0.05, healthy child self-report vs. caregiver proxy-report.

Table 5 lists the Pearson’s correlations coefficient calculated from analyses between Generic Core Scale and FIM Scale scores. Higher scores on every dimension of the Generic Core Scale were associated with higher scores on the FIM Scale (*p*<0.05).The physical, emotional, and social functioning dimension scores in the Generic Core Scale of the children’s self-reports correlated positively with scores on every dimension of the FIM Scale (*p*<0.05). Moreover, Generic Core Scale scores correlated positively with family communication scores from the FIM Scale (*p*<0.05).

**Table 5:** Pearson’s correlation coefficients (*r* values) for comparisons between Generic Core Scale and FIM Scale scores.

FIM Scale	Caregivers’ proxy-report Generic Core Scale					Children’s self-report Generic Core Scale				
	Physical	Emotional	Social	School	Summary	Physical	Emotional	Social	School	Summary
HRQOL	0.46^a^	0.59^a^	0.48^a^	0.53^a^	0.58^a^	0.51^a^	0.61^a^	0.58^a^	0.07	0.08
Physical	0.43^a^	0.58^a^	0.45^a^	0.50^a^	0.59^a^	0.57^a^	0.55^a^	0.51^a^	0.17	0.12
Emotional	0.40^a^	0.65^a^	0.48^a^	0.41^a^	0.59^a^	0.39^a^	0.54^a^	0.56^a^	0.07	0.12
Social	0.31^a^	0.53^a^	0.45^a^	0.44^a^	0.51^a^	0.49^a^	0.52^a^	0.56^a^	0.07	0.08
Cognitive	0.36^a^	0.45^a^	0.38^a^	0.54^a^	0.51^a^	0.45^a^	0.51^a^	0.44^a^	0.12	0.09
Communication	0.38^a^	0.44^a^	0.50^a^	0.33^a^	0.51^a^	0.24^a^	0.37^a^	0.42^a^	0.22	0.25^a^
Worry	0.20^a^	0.39^a^	0.35^a^	0.36^a^	0.37^a^	0.28^a^	0.47^a^	0.36^a^	0.01	0.14
Family Functioning	0.32^a^	0.45^a^	0.39^a^	0.37^a^	0.44^a^	0.32^a^	0.49^a^	0.47^a^	0.23	0.38
Daily Activities	0.24^a^	0.45^a^	0.39^a^	0.34^a^	0.42^a^	0.27^a^	0.47^a^	0.51^a^	0.00	0.10
Relationships	0.31^a^	0.47^a^	0.41^a^	0.37^a^	0.47^a^	0.29^a^	0.42^a^	0.36^a^	0.20	0.21
Summary score	0.41^a^	0.63^a^	0.53^a^	0.53^a^	0.63^a^	0.48^a^	0.61^a^	0.57^a^	0.14	0.17

a *p*<0.05.

Regression analysis s showed that a child’s age, relation to his/her caregiver, frequency of respiratory tract infections in the preceding year, caregiver’s education level, and caregiver’s HRQOL all significantly influenced a child’s summary score (*p*<0.05). As shown in Table 6, these variables differentially affected each dimension.

**Table 6:** Factors influencing dimensions of HRQOL in children with RRTIs.

Influence factors	Physical	Emotional	Social	School	Summary
Age (year)	0.13	–	–	–	1.08
Gender (male = 1,female = 2)	0.14	–	–	–	–
Relation between child and his/her caregiver (grandparents = 1,parents = 2)	–	–	–	–	2.04
Caregiver education level					
9–12 years	0.69	0.36	0.33	0.47	1.51
≥13 years	1.12	0.71	0.85	0.58	1.23
Caregiver’s HRQOL	–	0.31	0.32	–	0.36
Family economic status	–	−0.27	–	–	–
Frequency of upper RTIs in the preceding year	0.47	0.39	0.29	0.41	0.51
Frequency of lower RTIs in the preceding year	0.54	0.52	0.58	0.60	0.63
Determination coefficient *R* ^2^	0.69	0.43	0.51	0.34	0.76

Note: Family economic status was stratified according to the average monthly income per individual. For multiple regression analyses, 1 = ≤1500 yuan, 2 = 1500−3000 yuan, and 3 = ≥3000 yuan. RTIs indicated respiratory tract infection.

## Discussion

Our results showed that children with RRTIs displayed significantly lower HRQOL scores compared to healthy children. This result was based on physical, emotional, social, school functioning, and summary scales, and the two groups showed the greatest differences in Emotional functioning. In addition, RRTIs had a greater effect on the HRQOL of 5−7-year-olds compared to 2−4-year-old children. Moreover, families with RRTI-affected children scored lower on physical, emotional, social, cognitive functioning, communication, daily activities, relationships, economic status, and FIM summary scales scores compared to families with healthy children, however, RRTIs-affected families displayed greater Worry than families with healthy children. Taken together, these results demonstrate that RRTIs significantly influenced HRQOL in children, their caregivers, and their families.

The HRQOL assessment is becoming an important method to evaluate children’s physical and mental health, which highlights its importance and extensive application to pediatric clinical practice [16]. Studies investigating HRQOL among children with asthma, otitis, obesity, bacterial meningitis, and other diseases show that patient HRQOL scores are significantly lower than those of healthy control counterparts [17], [18]. Our study is the first to show that HRQOL is also decreased in patients with RRTIs compared to healthy children. Indeed, children with RRTIs scored lower than healthy, age-matched children on every dimension and summary scale of the PedsQL™ 4.0 Generic Core Scale. We also found that emotional functioning was the greatest dimension affected by RRTIs, and this finding is similar to the results of Sawyer et al [19], which suggested that emotional functioning is the dimension most affected by Asthma. Respiratory tract infections may lead to asthma, and our findings highlight the negative impact of these types of illnesses on the emotional functioning of pediatric patients.

Previous work by Upton et al [20] showed that parents of children in a nonclinical sample tend to report higher child HRQOL scores than the children themselves, while parents of children with health conditions tended to underestimate their children’s HRQOL. Similarly, caregiver proxy-reports show lower HRQOL in children with end stage renal disease [21] and cancer [22] than child self-reports. Likewise, the current data show that child self-report scores of HRQOL were higher than those of caregiver proxy-reports among children with RRTIs, which suggests that parents of children with RRTIs may underestimate their child’s HRQOL. We also found that healthy children’s self-reported HRQOL was significantly lower than that provided by their caregivers, although healthy children rated their emotional functioning significantly higher than their caregivers. These findings are consistent with the conclusion of Upton et al [20]. The assessment scale and dimensions may somewhat contribute to differences between patient and caregiver reports. For example, Cremeens et al [23] found low consistency between healthy child self-reports and parent proxy-reports on the PedsQL™ Generic Core Scale, with physical, psychosocial, and total scores for caregiver proxy-reports being higher than those of healthy child self-reports. One explanation for the discrepancy among HRQOL scores between caregivers and children may be that the degree of worry experienced by a caregiver is higher than that experienced by the ill child. Subsequently, worry and/or anxiety may influence a caregiver’s perception of their child’s health. Another explanation is that the comprehension ability of 5−7-year-old children is still developing, and their level of understanding and self-awareness may contribute to differences in the PedsQL™ 4.0 Generic Core Scale scores.

It is difficult to assess the accuracy of the results of child self-reports and caregiver proxy-reports. Children and caregivers may draw on different values and perspectives when they evaluate quality of life [24], [25]. Indeed, caregivers’ perceptions of children’s quality of life was not a convergent validation of the children’s views, but rather the caregivers independent viewpoints. Caregivers’ knowledge concerning their children’s disease-related experiences may be limited [25], particularly with respect to activities, events, or relationships that exist outside the home and with respect to internal emotional states, which have the potential to impact child’s social, school, and emotional well-being. These limitations might distort the results of caregiver proxy-reports. In addition, a chronic disease or disability may not affect the afflicted person’s quality of life in the same way as observed by others. For example, if patients have health problems, but are not bothered by their health constraints, their self-reporting on typical health status questionnaires may not reflect limitations due to their health issues [26]. Patients may wish to project their positive outlook by downplaying their health problems as less severe than a proxy rater. Based upon the possibility that the aforementioned psychological factors may influence participant scores in this study, we felt that both child self-reports and caregiver proxy-reports were important to HRQOL assessment. Child and caregiver results may be mutually complementary to HRQOL evaluation.Pediatric chronic health conditions and treatments not only impact children’s development, but they also influence the HRQOL of parents and family members [27]. For example, Hunfeld et al [28] reported that pediatric chronic pain has a significant, multi-domain impact on parental quality of life. Moreover, Jastrowski Mano et al [29] showed that FIM Scale scores associate positively with pain catastrophizing, functional disability, and emotional/behavioral problems, and are inversely related to pediatric quality of life. Sawyer et al [19] revealed that chronic disease has a significant negative effect on a patient’s family; however, the authors only assessed physical and emotional functioning of caregivers in this study. Our results showed positive correlations between the scale summary score and physical, emotional, and social dimensions, and a caregiver’s cognitive functioning, communication, worry, family daily activities, and family relationships. These results further confirm the influence of RRTIs on the family’s HRQOL.

Our current findings show that numerous variables influence HRQOL. For example, we found that a child’s age, the relation between child and his/her caregiver, a caregiver’s education level, and a caregiver’s own HRQOL were key factors contributing to the HRQOL in children with RRTIs. Similarly, Gerson’s [30] study on PedsQL of chronic kidney disease illustrates that higher maternal education level is associated with higher PedsQL scores on physical, school, and social dimension functioning. In addition, the younger the patient is, the worse the PedsQL score will be across the dimensions. Our data support this interpretation as we found that the higher a caregiver’s education level, the greater the HRQOL score. Moreover, older children with RRTIs reported better HRQOL than younger children.

The effects of medical interventions are reflected not only in the changes in somatic parameters but also in emotional and social aspects of the patients’ lives. Quality of life, which reflects well-being and functioning, is a relevant end-point for evaluating the efficacy of prevention measures, treatments, and rehabilitation in children [31]. Moreover, quality of life evaluations are critically informative toward developing clinical disease-management programs that provide comprehensive treatment and education for ill children and their families. Using an informed understanding of HRQOL as a foundation, pediatricians can develop targeted support services for children and their families, such as medical education about RRTIs and psychological counseling.

We believe our findings have important value regarding the quality of life of children affected by RRTIs. However, we acknowledge that our study has several major limitations. First, we were unable to obtain the exact duration of each RRTI episode and the lifetime RRTI duration because acceptable clinical tools needed to measure RRTIs severity in children are not currently available in China. Therefore, we could not evaluate the influence of these factors on HRQOL. However, the frequency of lower and upper RRTIs in the preceding year (the parameter we examined here) may reflect disease severity to some extent. Generally, infections of the lower respiratory system are more severe than infections of the upper respiratory system. Thus, we performed a multiple step-wise regression analysis that distinguished between upper and lower infections as an indirect assessment of intensity. Second, we did not objectively measure caregiver’s mental and physical state because we thought caregivers would have less influence on the children’s HRQOL than the children themselves. In this study, more than 60% of caregivers were parents, and less than 40% of caregivers were grandparents. Parents were fairly young, with a mean age of 30.6±2.1 years, which suggests that they were less likely to have chronic disease at this stage in their life. We assumed that grandparents who served as primary caregivers were likely in good health in order to take care of their grandchildren. An additional limitation to this study is that HRQOL results obtained from children at large AAA hospitals in Xi’an may not be representative of the HRQOL of patients treated at different hospitals across different regions. Although our subjects may not represent all children with RRTIs in China, or even in Xi’an, our data do provide significant insight into the effects of RRTIs on HRQOL, which is useful to clinicians and researchers.

### Conclusions

The existence of RRTIs was negatively associated with a child’s physical, emotional, social, and school functioning, and negatively affected caregivers’ quality of life, family daily activities, and relationships. Therefore, it is essential that we monitor quality of life measures in children with RRTIs and the health of their caregivers. We encourage health staff to increase education for caregivers, RRTI prevention, and psychological mediation in order to improve HRQOL in children with RRTIs and their affected family members.

## Acknowledgments

The research team is grateful to all the children and their families who participated in this study.
